# Permanent deconstruction of intracellular primary cilia in differentiating granule cell neurons

**DOI:** 10.1083/jcb.202404038

**Published:** 2024-08-13

**Authors:** Carolyn M. Ott, Sandii Constable, Tri M. Nguyen, Kevin White, Wei-Chung Allen Lee, Jennifer Lippincott-Schwartz, Saikat Mukhopadhyay

**Affiliations:** 1 Janelia Research Campus, Howard Hughes Medical Institute, Ashburn, VA, USA; 2 Department of Cell Biology, University of Texas Southwestern Medical Center, Dallas, TX, USA; 3 Department of Neurobiology, Harvard Medical School, Boston, MA, USA; 4 F.M. Kirby Neurobiology Center, Boston Children’s Hospital, Harvard Medical School, Boston, MA, USA

## Abstract

Primary cilia on granule cell neuron progenitors in the developing cerebellum detect sonic hedgehog to facilitate proliferation. Following differentiation, cerebellar granule cells become the most abundant neuronal cell type in the brain. While granule cell cilia are essential during early developmental stages, they become infrequent upon maturation. Here, we provide nanoscopic resolution of cilia in situ using large-scale electron microscopy volumes and immunostaining of mouse cerebella. In many granule cells, we found intracellular cilia, concealed from the external environment. Cilia were disassembled in differentiating granule cell neurons—in a process we call cilia deconstruction—distinct from premitotic cilia resorption in proliferating progenitors. In differentiating granule cells, cilia deconstruction involved unique disassembly intermediates, and, as maturation progressed, mother centriolar docking at the plasma membrane. Unlike ciliated neurons in other brain regions, our results show the deconstruction of concealed cilia in differentiating granule cells, which might prevent mitogenic hedgehog responsiveness. Ciliary deconstruction could be paradigmatic of cilia removal during differentiation in other tissues.

## Teaser

Before differentiating, cerebellar granule cell neurons require primary cilia to detect and proliferate in response to sonic hedgehog; however, mature granule cell neurons lack primary cilia. Here, Ott, Constable et al. describe in ultrastructural detail the pathway for postmitotic cilia deconstruction.

## Introduction

A singular, specialized, signal-detecting organelle, called the primary cilium, protrudes from the surface of most cells including neurons (Green and Mykytyn, 2014; Louvi and Grove, 2011; Ott et al., 2024; Rosenbaum and Witman, 2002; Wu et al., 2024). Receptors and effectors compartmentalized in the cilium mediate efficient detection and transduction of external signals (Anvarian et al., 2019; Nachury and Mick, 2019). Even deep in tissues, cilia perceive and respond to signals that can initiate developmental programs or alter cell activity. Despite the significant functions of cilia, variations in cilia structure within tissues have just begun to be studied (Mill et al., 2023). In the brain and spinal cord, for example, cilia abundance and length can change (Das and Storey, 2014; Tu et al., 2023). Because cilia sense growth factors, neuropeptides, and neuromodulators, changes in cilia can impact neurodevelopment, neuronal function, neuronal circuit connectivity, neuronal excitability, and neuropathology (Bowie and Goetz, 2020; Guo et al., 2017; Higginbotham et al., 2012; Kumamoto et al., 2012; Lee and Gleeson, 2011; Sheu et al., 2022; Suciu and Caspary, 2021; Tereshko et al., 2021; Wilsch-Brauninger and Huttner, 2021; Youn and Han, 2018). To understand these processes, we need to understand how changes in cilia ultrastructure contribute to neurodevelopmental programs, especially in intact tissue.

Approximately 80% of adult human brain neurons are packed into the inner layer of the cerebellum (Herculano-Houzel, 2009). These neurons are a singular type of neuron called granule cells (GCs). GC neurons are particularly interesting from a cilia perspective because their expansion from immature progenitors occurs in response to sonic hedgehog (SHH), a soluble ligand detected by receptors in the ciliary membrane, which triggers progenitor proliferation (Dahmane and Ruiz i Altaba, 1999; Wallace, 1999; Wechsler-Reya and Scott, 1999). Primary cilia are necessary to maintain the proliferative state of progenitors, and mutations that disrupt ciliogenesis lead to cerebellar hypoplasia and abnormalities in foliation (Chizhikov et al., 2007; Spassky et al., 2008). Upon onset of differentiation, GCs stop responding to SHH, begin to extend axons, and migrate along glial processes toward Purkinje neuron cell bodies (Rakic, 1971). During differentiation, reductions in cilia length and frequency have been reported (Chang et al., 2019; Di Pietro et al., 2017; Ong et al., 2020), and in adult GCs, staining revealed only occasional cilia (Di Pietro et al., 2017). These data indicate that cilia loss occurs in postmitotic, differentiating granule cell neurons. Cilia disassembly in other systems, however, has only been rigorously investigated during premitotic cilia resorption (Pan et al., 2004; Pugacheva et al., 2007; Tucker et al., 1979). Detailed ultrastructural analysis of cilia disassembly in the context of developing tissue has been challenging because cilia and centrosomes are small, singular structures.

To characterize morphological changes in cilia within the cerebellum during neurogenesis, we mapped and quantified centrosomes and cilia in hundreds of GCs using large-volume electron microscopy (EM) datasets of adult and developing mouse cerebella (Nguyen et al., 2023; Wilson et al., 2019). By combining high-resolution EM with the molecular specificity of immunofluorescence imaging, we quantified modulation in cilia length and abundance, obtaining unprecedented nanoscopic images of centrosomes and cilia across the span of neurogenesis. Our findings reveal a process for cilia loss during GC differentiation that we call cilia deconstruction. We report surprising intermediates and end states in the ciliary deconstruction process. In mature GCs, centriole distal appendages were anchored to the plasma membrane without forming cilia. Together, the results demonstrate for the first time dynamic cilia architecture at the nanoscale across tissue and offer insights into cilia maintenance and modulation pathways, likely relevant to other tissues during development.

## Results

### Primary cilia length and frequency are decreased during GC neurogenesis

**Figure 1.**
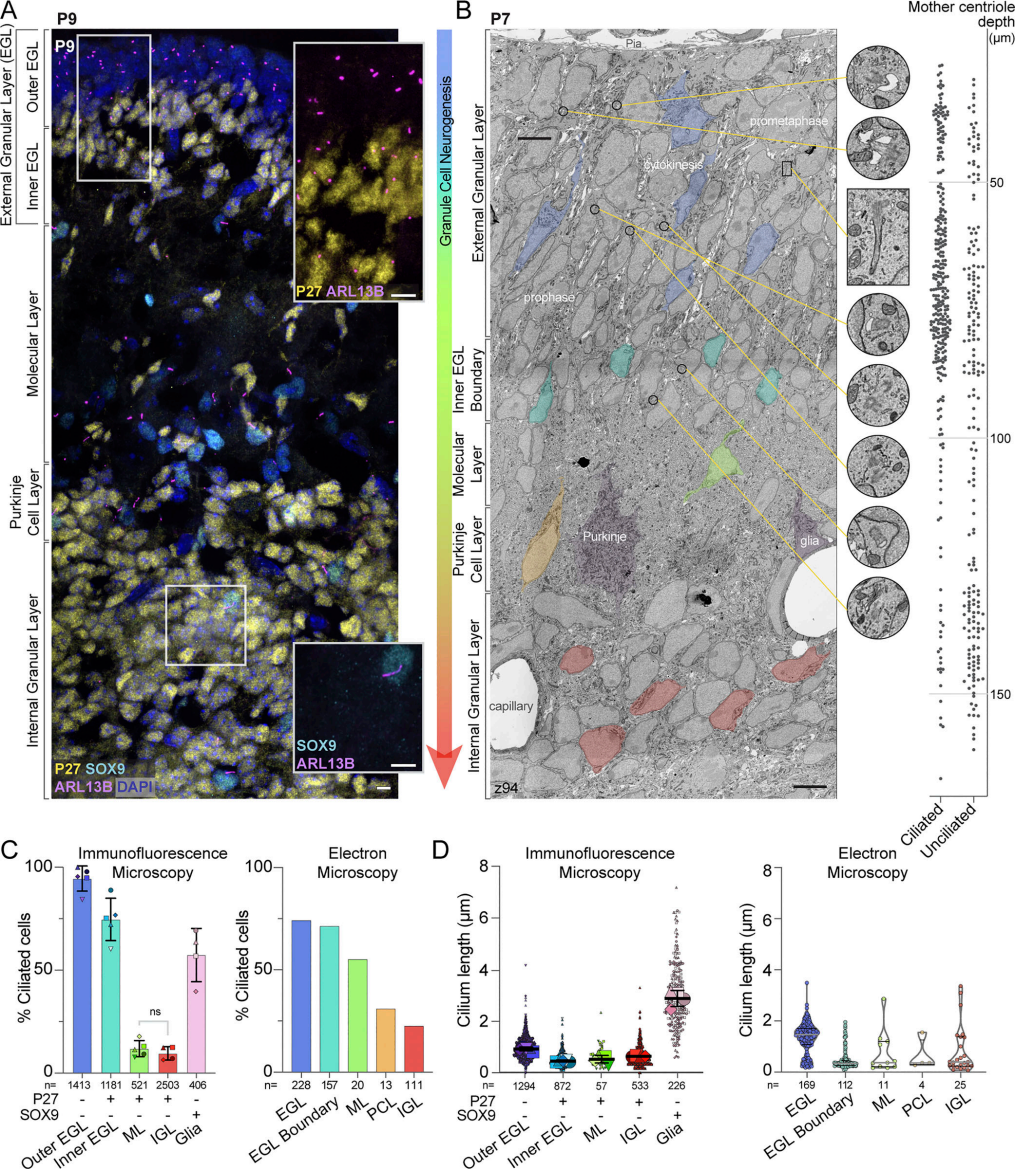
**Differentiating GCs initially have short cilia that are lost as neurons mature.** **(A)** A sagittal section of P9 mouse cerebellum immunostained with antibodies to the GC differentiation marker P27^KIP^ (yellow), the cilia marker ARL13B (magenta), the glial marker SOX9 (cyan), and counterstained with DAPI (blue) then imaged with spinning disc confocal microscopy. The layers of the developing cerebellum are indicated on the left. Scale bar: 10 μm. **(B)** A single slice of the P7 large serial-section scanning EM volume with GCs in different stages of differentiation is highlighted. The phase of mitotic cells in the EGL are superimposed on the GCs. In addition, cropped images of centrosomes and cilia from the panel are magnified and the location of each image is indicated by a yellow line. On the right side of the image, the depth of each ciliated and unciliated mother centriole is plotted. Scale bar: 5 μm; diameter of zoom regions: 1.6 μm. **(C)** Cilia frequency is quantified from measurements of widefield immunofluorescent images (left; three sections from each of four or five animals) and from each annotated mother centriole in the P7 serial scanning EM volume (right). Differentiating cells in the immunofluorescent images were identified based on the expression of P27^KIP^. Because the EM volumes lack molecular markers, we instead used cellular context and identified GCs near the EGL boundary as a pool of differentiating cells. Glia were included in the immunofluorescent analysis because SOX9 staining allowed us to distinguish them with confidence. **(D)** The length of each cilium from the widefield images is plotted on the left where each individual cilium measurement is represented by a small symbol and the average for each animal is represented by a larger symbol. The line and error bars represent the mean and standard deviation of the individual animal averages. The measured length of each cilium annotated in the P7 EM volume is quantified on the right.

Reductions in both cilia length and frequency during granule cell neurogenesis have been reported previously (Chang et al., 2019; Di Pietro et al., 2017; Ong et al., 2020). To identify when in neurogenesis and where in the postnatal developing cerebellum cilia disassembly occurs, we quantified cilia status throughout the entire progression of GC differentiation. Cerebellar sections from postnatal day 9 (P9) mice were immunostained and imaged using an antibody to ARL13B (Fig. 1 A), an atypical GTPase that is highly enriched in cilia (Caspary et al., 2007; Duldulao et al., 2009). At the same time, we stained for SOX9 to identify glia (Farmer et al., 2016; Sun et al., 2017; Vong et al., 2015) and the cell cycle inhibitor P27^KIP1^ to delineate differentiating and mature GCs from cycling GC progenitors (Miyazawa et al., 2000). The progression of GC neuronal maturation coincides with migration deeper into the tissue. Therefore, in a single sagittal section, the maturation state of each P27^KIP1^ expressing GC can be inferred by the depth of the GC in the cerebellum (Leto et al., 2016). As expected, GC progenitors that populated the external granule layer (EGL) of the emerging cerebellum lacked P27^KIP1^ expression, while GCs newly committed to differentiation that expressed P27^KIP1^ had exited the proliferation zone and were located in the inner EGL (Fig. 1 A). Differentiating GCs migrating through the molecular layer (ML), past the Purkinje soma in the Purkinje cell layer (PCL), were also expressing P27^KIP1^, as were GC neurons that were located in the internal granular layer (IGL) in the latest stages of neurogenesis and maturation (Fig. 1 A). ARL13B staining was used to measure both cilia frequency and length throughout the cerebellar layers. We found that in the outer EGL, ∼94% of the GC progenitors were ARL13B positive, indicating they had a cilium (Fig. 1, A and C). The fraction of ciliated cells decreased to 75% in the P27^KIP1^-positive differentiating cells in the inner EGL. Cells that had migrated beyond the EGL had dramatically fewer cilia: only 12% were ciliated in the ML and 10% in the IGL (Fig. 1, A and C). We also compared the length of cilia in each layer of the developing cerebellum (Fig. 1 D). The GC progenitor cilium averaged around 1 μm, while cilia in differentiating GCs were shorter. By contrast, glial cells (i.e., SOX9 positive cells) in the IGL layer had a mean cilium length of ∼3 μm (Fig. 1, C and D). Because cilia were frequent but shorter in cells that had just begun differentiating and then became rare as GC neurons matured, we concluded that the short cilia on cells in the inner EGL disassemble, typically before GCs migrate into the ML.

Neuronal cilia in other brain regions show heterogeneity with respect to ciliary membrane composition and identification using markers such as ARL13B (Kasahara et al., 2014; Sipos et al., 2018). To ensure that changes in cilia immunoreactivity were not being misinterpreted as cilia loss, we analyzed cilia imaged by volumetric EM. The improved resolution of EM enabled us to obtain detailed views of cilia ultrastructure as well as more accurate cilia measurements (many cilia lengths were close to the resolution limits of light microscopy, especially in z). For this investigation, we uploaded, annotated, and analyzed centrosomes and cilia in GCs of the published 1.7 × 10^6^ μm^3^ (2,513 z slices) serial scanning EM volume of a P7 mouse cerebellum (Wilson et al., 2019), which has 4 nm resolution in x and y and 30 nm resolution in z. The improved resolution in the EM data made it possible to identify the migrating GCs in the PCL which were grouped with the ML in the immunofluorescence analysis (Fig. 1 B). Inferring a pseudotimeline of GC differentiation was slightly different without a molecular marker like P27^KIP1^. We could not discern the boundary between proliferating GC progenitors and GCs newly committed to differentiation (i.e., distinguish outer from inner EGL). However, we knew that all GCs that had reached the inner edge stained with P27^KIP1^ (Fig. 1 A), so we annotated the GCs at the “EGL boundary” if its cell body, or the cell body next to it, was directly adjacent to the ML (Fig. 1 B). Thus, in the EM data, we could compare GCs classified as EGL, which included both proliferating and early differentiating GCs, to GCs at the EGL boundary, which were all differentiating neurons.

To evaluate cilia length and frequency modulation with the improved accuracy possible from EM, we traced and annotated cilia throughout the layers of the developing cerebellum. Detailed measurements for each cilium are included in Table S1. We quantified the GC cilia frequency and length measured in each layer of the EM volume and compared the results to measurements from light microscopy. Overall, the cilia distributions were remarkably similar (Fig. 1, C and D). It is likely that the measured frequency of cilia in the EGL differs between the two measurements because the EGL in the EM data includes cells in both the inner and outer EGL. Cilia frequency was decreased in cells that had migrated to the ML, the PCL, and the IGL. When compared with the rest of the EGL, the GCs in the EGL boundary had similar cilia frequency, however, the median cilium length was much shorter (0.3 μm in the EGL boundary compared to 1.5 μm in the EGL). The ML and PCL in the EM dataset had higher cilia frequency than quantified in the ML by immunofluorescence. These differences could be related to sample size differences and/or the 2-day age difference between the animals imaged. Previous EM analysis of cilia in the IGL reported ∼40% ciliation in one P6 mouse with *n* = 8 cells (Chang et al., 2019). We detected cilia in 23% of IGL GCs in one P7 mouse by EM and in 6–12% of IGL GCs in four mice by IF at P9. The differences could be due to individual animal variation and/or maturation during aging. In our EM analysis, most of the cilia were short (10 of 25 cilia were <300 nm), however, two cilia in the IGL were >3 μm (Fig. 1 D). Many arriving immature GCs can lack synapses in the IGL, which can make it difficult to discriminate from glia/astrocytes in EM images. Therefore, we suspect these cilia belong to velate astrocytes (Farmer et al., 2016; Sun et al., 2017; Vong et al., 2015), which our immunofluorescence studies using glia molecular markers showed had significantly longer cilia than GCs in the IGL (Fig. 1 D). Overall, the EM and IF analysis indicate that cilia length decreased before cilia disassembled.

**Figure S1.**
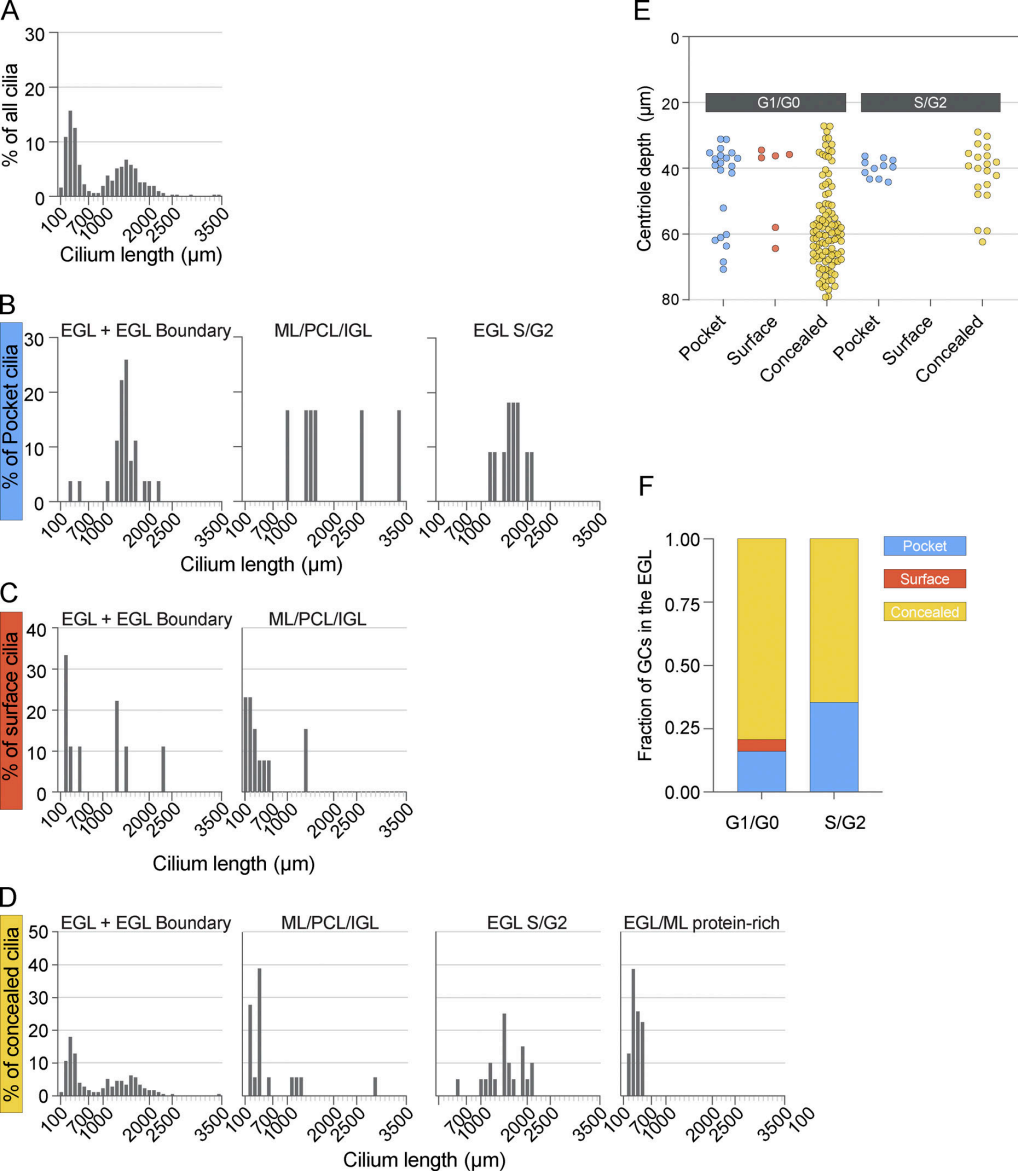
**Bimodal distribution of cilia lengths. (A)** Cilia from the P7 volume were binned by 100 nm and the distribution of cilia lengths was plotted as a percentile of total cilia. **(B–D)** The distribution of cilia lengths plotted in A is separated by pocket (B), surface (C), and concealed (D) cilia types. Within each cilium type, the distributions are shown by cell layer with S/G2 cilia and protein-rich cilia plotted separately. There are no S/G2 cells with surface cilia and all protein-rich cilia are concealed. **(E)** The depth of the basal body for each cilium in the EGL is plotted based on cilium type. Cells in G1/G0 are on the left and cells in S/G2 are on the right. **(F)** The pocket, surface and concealed cilia in the EGL are plotted as a fraction of the total G1/G0 cells and S/G2 cells. Cells in the EGL boundary were excluded from E and F.

To assess the heterogeneity of cilia length, we graphed the distribution of cilia lengths from all GCs in the EM dataset (Fig. S1 A). We observed a bimodal distribution indicating two populations of cilia. Short cilia peaked around 300 nm and were all <750 nm. The distribution of longer cilia ranged from 1 to 2 μm. Long cilia were predominantly found in progenitors and cilia were shorter in newly differentiating GCs.

### Cilia on differentiating GCs are assembled post-mitotically and then disassemble

**Figure 2.**
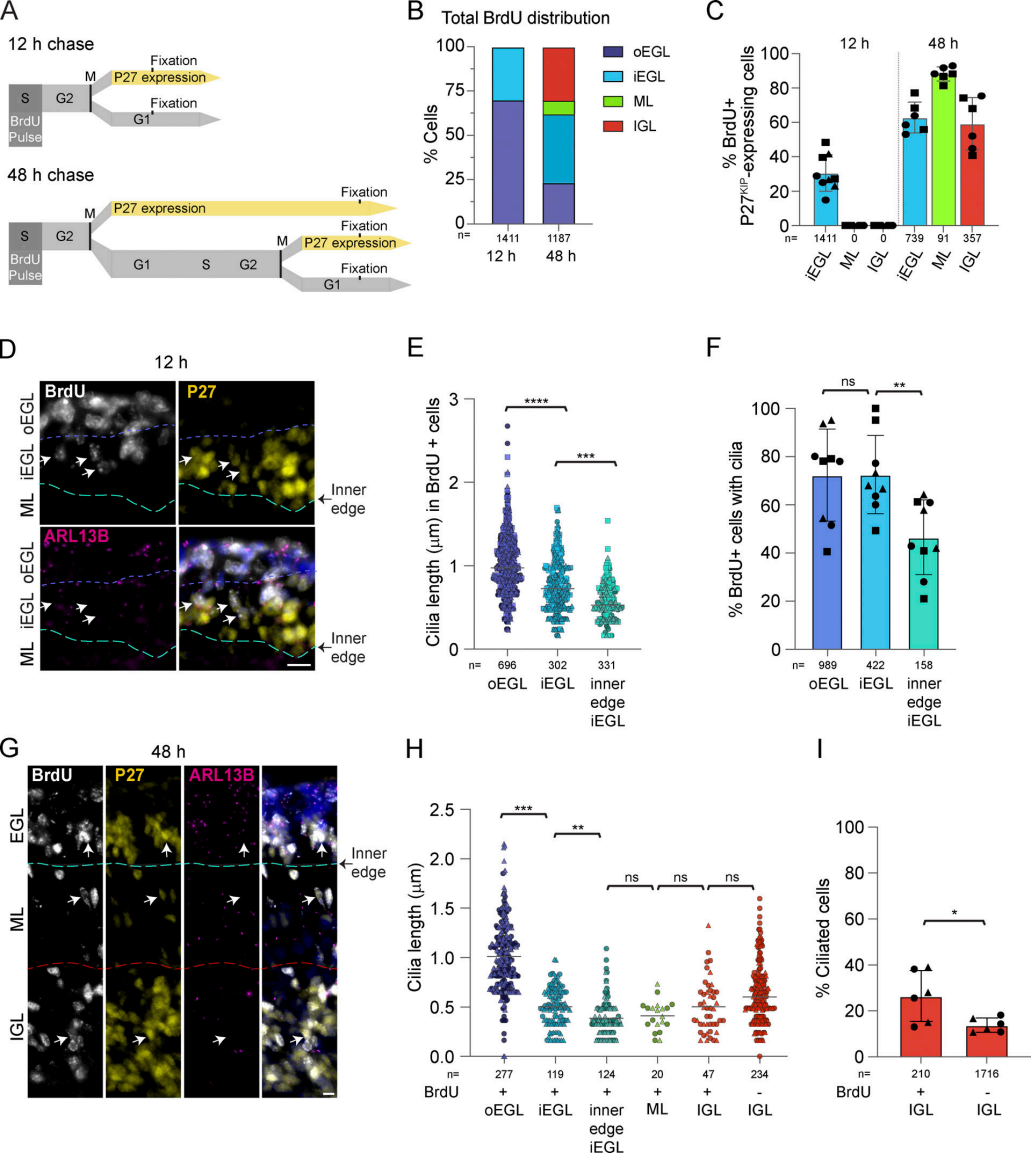
**BrdU labeling reveals that early differentiating GCs have short primary cilia that subsequently disassemble. (A)** P7 mice were injected with the thymidine analogue BrdU and cerebella were harvested after 12 or 48 h. BrdU incorporates into the DNA of cycling cells during the S phase. BrdU-labeled cells that began differentiating became P27^KIP^ positive and migrated deeper. **(B)** BrdU-labeled cells were classified by location in the developing cerebellum. The distribution of BrdU-labeled GCs was quantified 12 and 48 h after BrdU injection. **(C)** The percentages of total BrdU labeled cells in each layer that expressed P27^KIP^ 12 and 48 h after BrdU injection were quantified from three sections from two to three animals. Shapes denote biological replicates. **(D)** Sagittal sections of P7 mouse cerebella harvested 12 h after BrdU injection were immunostained with antibodies to BrdU (white), P27^KIP^ (yellow), ARL13B (magenta), and counterstained with Hoechst (blue in merge panel) before imaging with widefield microscopy. The approximate boundaries between layers are indicated in white and yellow. Arrows denote BrdU labeled P27^KIP^ positive cells. Scale bar: 10 μm. **(E)** BrdU-labeled ciliated cells in the inner and outer EGL were distinguished based on P27^KIP^ expression and cells at the inner edge of the EGL were directly adjacent to the ML. The measured lengths of cilia in these cells are plotted. *n* = number of cilia counted. **(F)** The percentages of BrdU-positive GCs with cilia in the outer EGL (P27^KIP^ negative), the inner EGL (P27^KIP^ positive), and at the inner edge (P27^KIP^ positive) were quantified 12 h after BrdU administration. **(G)** Sagittal sections of P7 mice cerebella harvested 48 h after BrdU injection were immunostained with antibodies to BrdU (white), P27^KIP^ (yellow), and ARL13B (magenta), and counter-stained with Hoechst (blue in merge panel) before imaging using widefield microscopy. The approximate boundaries between layers are indicated in yellow. Arrows denote BrdU labeled P27^KIP^ positive cells. Scale bar: 10 μm. **(H)** The length of each cilium in the indicated layers 48 h after BrdU labeling is plotted. EGL layers were demarcated by P27^KIP^ as in F. **(I)** The percentage of ciliated GCs in the IGL is plotted for BrdU labeled and unlabeled cells 48 h after injection. P values: 0.0332 (*), 0.0021 (**), 0.0002 (***), <0.0001 (****).

To further investigate the timing and location of cilia disassembly during differentiation, we evaluated cilia length and frequency in a subpopulation of GCs that had just begun differentiating. To do this, we injected P5 or P7 pups with BrdU and harvested the developing cerebella after either 12 or 48 h. BrdU is a thymidine analog incorporated into DNA during the S phase. Based on cell cycle dynamics in GC progenitors (Espinosa and Luo, 2008; Ho et al., 2020), differentiating GCs immediately exiting the cell cycle would be captured by the 12-h pulse chasing. The 48-h chase permitted another round of division for cycling GC progenitors but also captured postmitotic GCs that began differentiating and migrating immediately after the BrdU pulse (Fig. 2 A). We imaged the sections after staining for BrdU, P27^KIP1^, and ARL13B. The BrdU-labeled cells were restricted to the EGL in animals with a 12-h chase but more than a third had moved out of the EGL after a 48 h chase (Fig. 2 B). We also quantified the location of recently divided cells that had stopped proliferating and committed to differentiation by measuring BrdU-labeled cells that also expressed P27^KIP1^. By 48 h, the majority of BrdU-labeled cells in all layers also expressed P27^KIP1^ (Fig. 2 C). We concluded that these subpopulations of recently divided GCs could be used to gain more insights into cilia changes during neuronal differentiation.

To determine whether cilia shorten and/or disassemble as GCs progress through differentiation, we compared the cilium length and frequency at different stages of differentiation based on the migration through the cerebellar layers. GC cilia in the P27^KIP1^-expressing, BrdU-positive cells of the inner EGL were shorter than the BrdU-labeled progenitor cells in the outer EGL (0.7 and 1 μm respectively; Fig. 2, D–E). The BrdU and P27^KIP1^ positive cells directly at the inner edge of the EGL, poised to migrate across the ML, were shorter (0.5 μm) and less frequent than the P27^KIP1^-expressing, BrdU-positive cells of the inner EGL that had not migrated as far (Fig. 2, E and F). Similar differences in cilia length and frequency were measured 48 h after BrdU treatment (Fig. 2, G and H). These results indicate that the small cilia shorten before arriving at the EGL boundary.

Although most GCs have no detectable cilium after migrating beyond the EGL, a small number of GCs in the IGL were ciliated. To determine whether cilia disassembly can continue outside the EGL, we used the BrdU labeling to distinguish between newly arrived GCs and more mature neurons. Specifically, cells that express P27^KIP1^ but do not stain with BrdU arrived in the IGL before the BrdU-positive GCs. Cilia lengths in the two populations were comparable (Fig. 2 H). However, we found that the cilia frequency was higher for BrdU-positive cells in the IGL (Fig. 2 I). Together, these data indicate that cilia in differentiating GCs shorten in the inner EGL and that cilia continue to be disassembled in maturing GCs in the IGL.

### Cilia disassembly in progenitor GCs is distinct from disassembly in differentiating GCs

**Figure 3.**
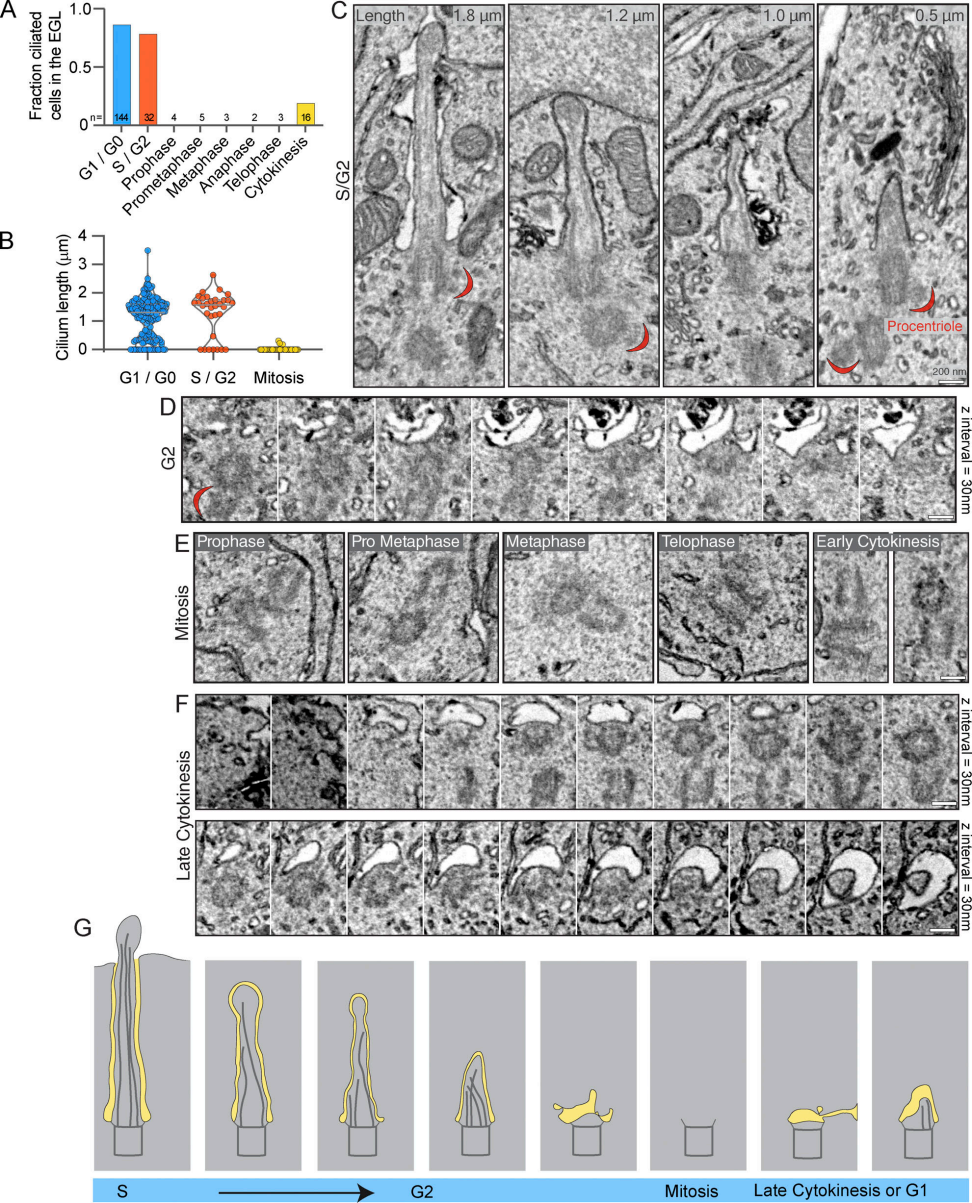
**Cilia are completely resorbed during mitosis in GC progenitors. (A)** The cell cycle status of each cell in the EGL of the P7 SEM volume was determined and annotated. The fraction of cells in each phase with primary cilia is quantified. **(B)** The length of each EGL GC cilium is plotted by cell cycle phase. G1 and G0 cells cannot be distinguished; both have two centrioles. **(C)** Serial scanning EM images of cilia from four individual cells with duplicating centrioles that indicate the cells are in S phase or G2. Cilium length is noted in the upper right corner and procentrioles visible in the same section are indicated with a red crescent. **(D)** Serial sections of a non-ciliated mother centriole and procentriole in a G2 cell in the EGL. **(E)** Representative SEM images of non-ciliated centrioles in GC progenitors in each stage of mitosis. **(F)** Serial sections of mother centrioles from late-stage cytokinesis cells with a ciliary vesicle (upper) or nascent cilium (lower). **(G)** A model of the process of cilia resorption using representations of the cilia presented in C–F. Prior to mitosis, cilia disassemble, are absent during mitosis, and reappear late in cytokinesis.

Cilia on postmitotic GCs at the EGL boundary were <750 nm (Fig. 1 D). This population of short cilia is unlikely to arise from the gradual shortening of longer cilia in the outer EGL because cilia length distribution in GCs was not continuous but was bimodal, peaking around ∼0.5 and ∼1.5 μm, with very few intermediate length cilia (Fig. S1 A). Light microscopy has shown that cultured mouse medulloblastoma GCs resorb cilia prior to mitosis in vitro (Ho et al., 2020). There are thus two ways cilia disassembly during progenitor cell division could have generated the population of short cilia: (1) each cilium was resorbed prior to GC progenitor mitosis and cilium regrowth was stunted in differentiating daughter cells; or (2) short cilia were remnants of progenitor cilia retained through mitosis as has been observed in other neural progenitors (Paridaen et al., 2013). To determine if any remnants of cilia remain associated with mother centrioles during mitosis in situ, we examined centrioles in dividing progenitor cells in the P7 EM volume. We first identified progenitor cells in S-phase, G2, and mitosis. Procentrioles were visible in the EGL templating off the mother and daughter centrioles indicating the cells were in S phase and G2 (Fig. 3 C) (Kumar and Reiter, 2021; O'Toole and Dutcher, 2014). Although some procentrioles could be classified as nascent or mature, the distinction was sometimes complicated by cut angle and imaging artifacts; so for the analysis, we grouped all cells with a procentriole into a combined S/G2 category. We found cilia in most S/G2 progenitor cells (Fig. 3, A–C), indicating that cilia disassembly occurs just before cells enter mitosis. This is similar to results in cultured GC progenitors and other cells, where cilia disassemble sometime after reaching S phase (Ford et al., 2018; Ho et al., 2020). Generally, cilia length in S/G2 cells was similar to cells in G1/G0 (Fig. 3, B and C). Cilia disassembly appeared to involve internalized cilia and, at the latest stage, centriole-associated membranes (Fig. 3, C and D). Cilia disassembly was completed by the time GCs entered mitosis. We found no axonemes or membrane vesicles associated with mitotic centrioles (Fig. 3, A and E), which differs from the conclusion that cilia persist into mitosis in cultured GC progenitors (Ong et al., 2020). The early ciliogenesis intermediates shown in Fig. 3 F were observed only in GCs in which cytokinesis had progressed sufficiently that midbody constriction was evident and the cytokinetic bridge was largely enveloped within a daughter cell. These observations indicate that cilia are completely disassembled prior to mitosis and ciliary remnants are not carried through into differentiating GCs. In addition, the disassembly of short cilia during GC differentiation occurs in postmitotic cells and is thus distinct from premitotic cilia disassembly. To distinguish the processes, we will refer to the novel dismantling of cilia in post-mitotic GCs as cilia deconstruction.

### Most GC cilia are intracellular and can be concealed from the external environment

**Figure 4.**
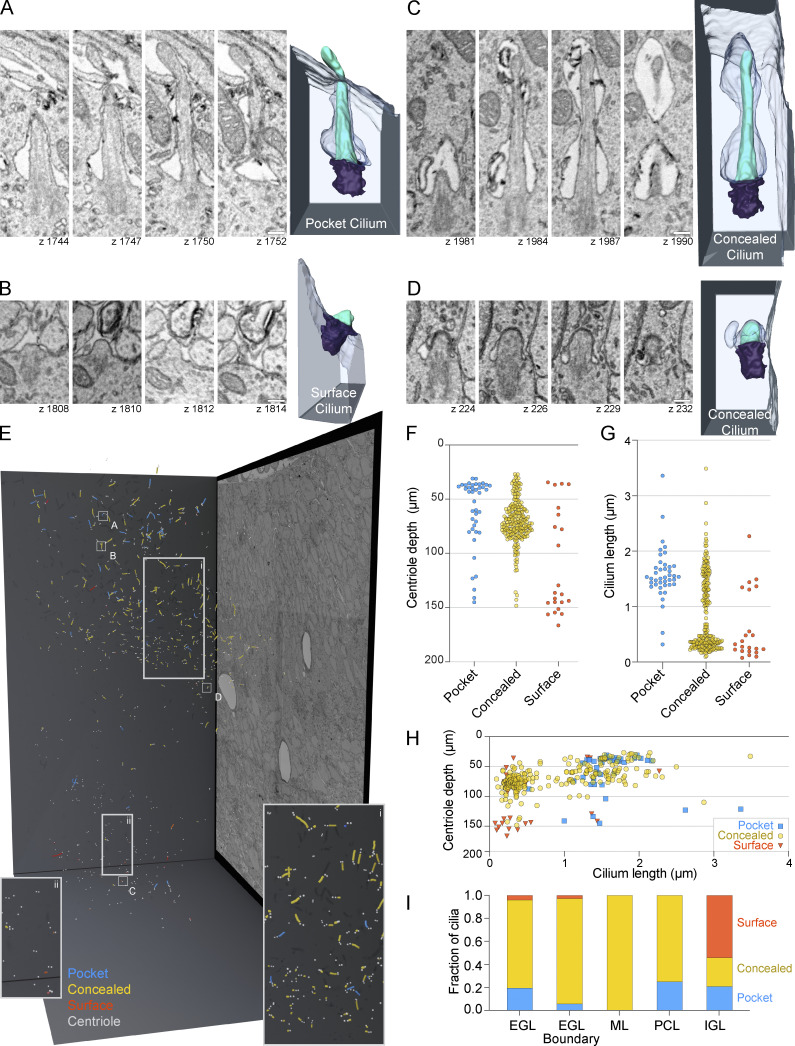
**Many GC cilia are submerged from the cell surface and concealed intracellularly. (A–D)** Representative pocket (A), surface (B), and concealed (C and D) cilia from P7 large serial scanning EM volume are displayed. Select z slices of each cilium are presented to the right of the 3D reconstructed image of each cilium. Scale bar: 200 nm. **(E)** Annotated cilia and centrioles are represented in the 3D space of the serial scanning EM volume. Annotated centrioles are represented as gray spheres and cilia as lines. Cilia color represents the classification of cilia as Pocket (blue), Concealed (yellow), or Surface (red). The positions of the cilia displayed in A–D are indicated. Insets show regions marked (i) and (ii). **(F)** The depth of mother centrioles is plotted for each type of cilium. In F and H, the y-axis is inverted such that higher depth values fall farther below the Y-axis. **(G)** Cilium length is plotted for each type of cilium. **(H)** The length of each annotated cilium is plotted as a function of mother centriole depth. The cilia are color coded as classified. **(I)** The fractional distribution of cilia in each layer is plotted and colored according to cilia type.

**Figure S2.**
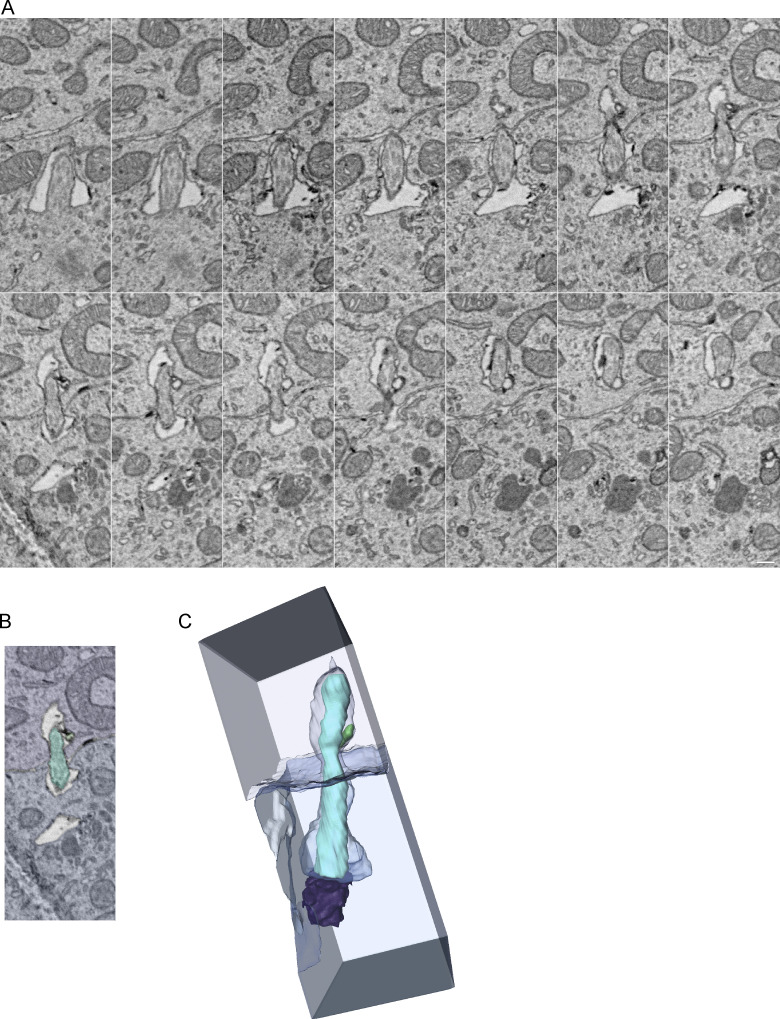
**Cilium is enveloped by an adjacent cell. (A)** Serial EM sections of a pocket cilium that exits the cell in the bottom half of the image and is enveloped by the adjacent cell. Scale bar is 200 nm and z interval is 30 nm. **(B)** Single z slice from the sections in A colored to highlight the cilium (cyan), the cell of cilium origin (blue), and the enveloping cell (lavender). **(C)** 3D reconstruction of the same cilium. The basal body is purple, and the membrane inclusion adjacent to the cilium is green.

We next examined the ultrastructure of cilia within the volumetric EM to gain insights into differences between long and short cilia and in this process discovered that external exposure of cilia was also modulated. While cilia in some cells protrude directly from centrioles docked at the cell surface, most cilia that extend from centrioles deeper in the cell are ensheathed in a membranous pocket, called a ciliary pocket that invaginates from the cell surface and anchors at the distal appendages (Benmerah, 2013; Rohatgi and Snell, 2010). As has been seen in published images (Chang et al., 2019; Ong et al., 2020; Spassky et al., 2008), many progenitor GC cilia had ciliary pockets (Fig. 4 A and Video 1). These “pocket cilia” were largely ensheathed along the cilium length and often only the cilium tip extended into the extracellular space (Fig. 4 A). Upon exiting the pocket over 25% of pocket cilia were enveloped in a plasma membrane invagination of an adjacent cell (Fig. S2). We observed a second class of cilia, “surface cilia,” that lacked a ciliary pocket (Fig. 4 B and Video 2). The third and largest group of cilia were contained in an ensheathing membrane—submerged in the cytoplasm (Fig. 4, C and D; and Videos 3 and 4). We refer to these as “concealed cilia.” The only potential access concealed GC cilia had to the extracellular space was through tubules or pores that resemble the dynamic tubules between the ensheathing membrane and the cell surface during ciliogenesis in cultured cells (Ganga et al., 2021; Insinna et al., 2019; Stuck et al., 2021). We conclude that with the exception of surface cilia, membrane structures were positioned to modulate cilia exposure to extracellular milieu.

**Video 1.** **Pocket cilium in the EGL.** This z series includes the entire pocket cilium of cell 451 from the EGL of the P7 cerebellum shown in Fig. 4 A. 5 frames per second.

**Video 2.** **Surface cilium in the IGL.** This z series includes the entire surface cilium of cell 447 from the IGL of the P7 cerebellum shown in Fig. 4 B. 5 frames per second.

**Video 3.** **Concealed cilium in the EGL.** This z series includes the entire concealed cilium of cell 518 from the EGL of the P7 cerebellum shown in Fig. 4 C. 5 frames per second.

**Video 4.** **Concealed cilium in the EGL boundary.** This z series includes the entire pocket cilium of cell 368 from the EGL boundary of the P7 cerebellum shown in Fig. 4 D. 5 frames per second.

To determine whether any of the ciliary ultrastructures were enriched at specific developmental stages, we examined the distribution of pocket, surface, and concealed cilia within the developing cerebellum. A 3D projection of all cilia by type is displayed in Fig. 4 E. The distributions of each cilium type by centriole depth (distance from pia), cilium length, and layer are quantified in Fig. 4, F–H and are shown with respect to the cell cycle in Fig. S1, E and F. Concealed cilia were dominant in the EGL, both in G1/G0 and S/G2 cells. Pocket cilia were generally >1 μm and most were found in the outer EGL indicating that they were present on GC progenitors. Overwhelmingly, the cilia at the EGL boundary were both short and concealed. Surface cilia, while occasionally observed in the EGL, were the dominant cilium type in the IGL. Because cilia deconstruction occurred in both the EGL boundary and IGL, we conclude that the disassembling cilia during GC differentiation were most likely concealed and surface cilia.

### Large-scale EM reveals novel cilia disassembly intermediates associated with maturing GCs

**Figure 5.**
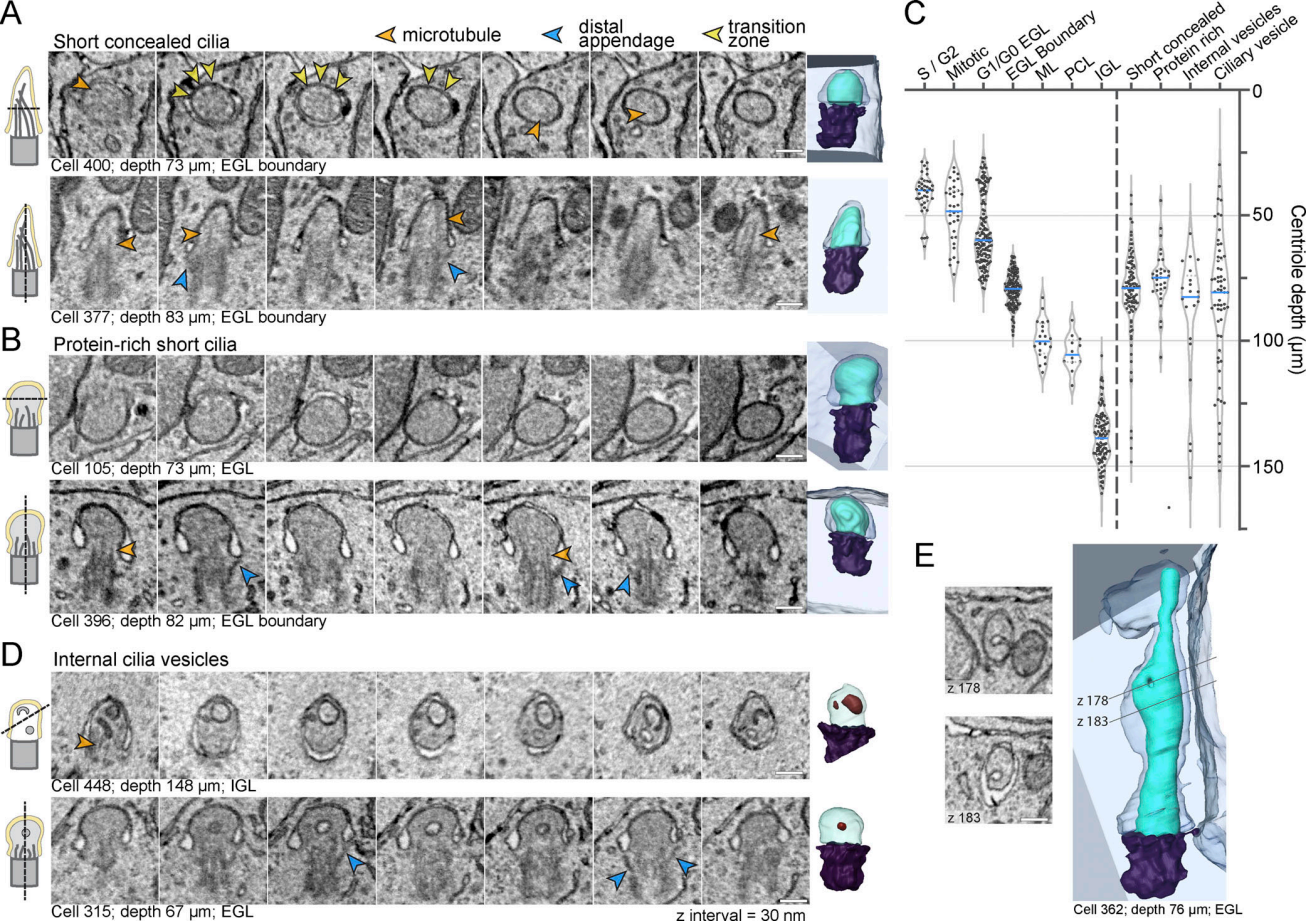
**Large-scale EM reveals novel cilia disassembly intermediates in differentiating GCs. (A, B, and D)** Serial sections of representative short, concealed cilia (A), protein-rich short cilia (B), and cilia with internal vesicles (D) are displayed. The section orientation is indicated by the cartoon on the left and the 3D reconstructions of each cilium is displayed on the far right. **(C)** Mother centriole depth is plotted on an inverted y-axis such that higher depth values fall farther below the y-axis. To the left of the dotted vertical line, the centrioles are classified by layer, with the outer EGL the cells broken out by cell cycle phase. On the right side of the dashed line, the mother centrioles are grouped based on the type of cilia deconstruction intermediates observed. **(E)** Two representative EM sections showing invagination of the ciliary membrane are shown to the left of the 3D reconstruction of the concealed cilium from the EGL. All scale bars are 200 nm.

**Figure S3.**
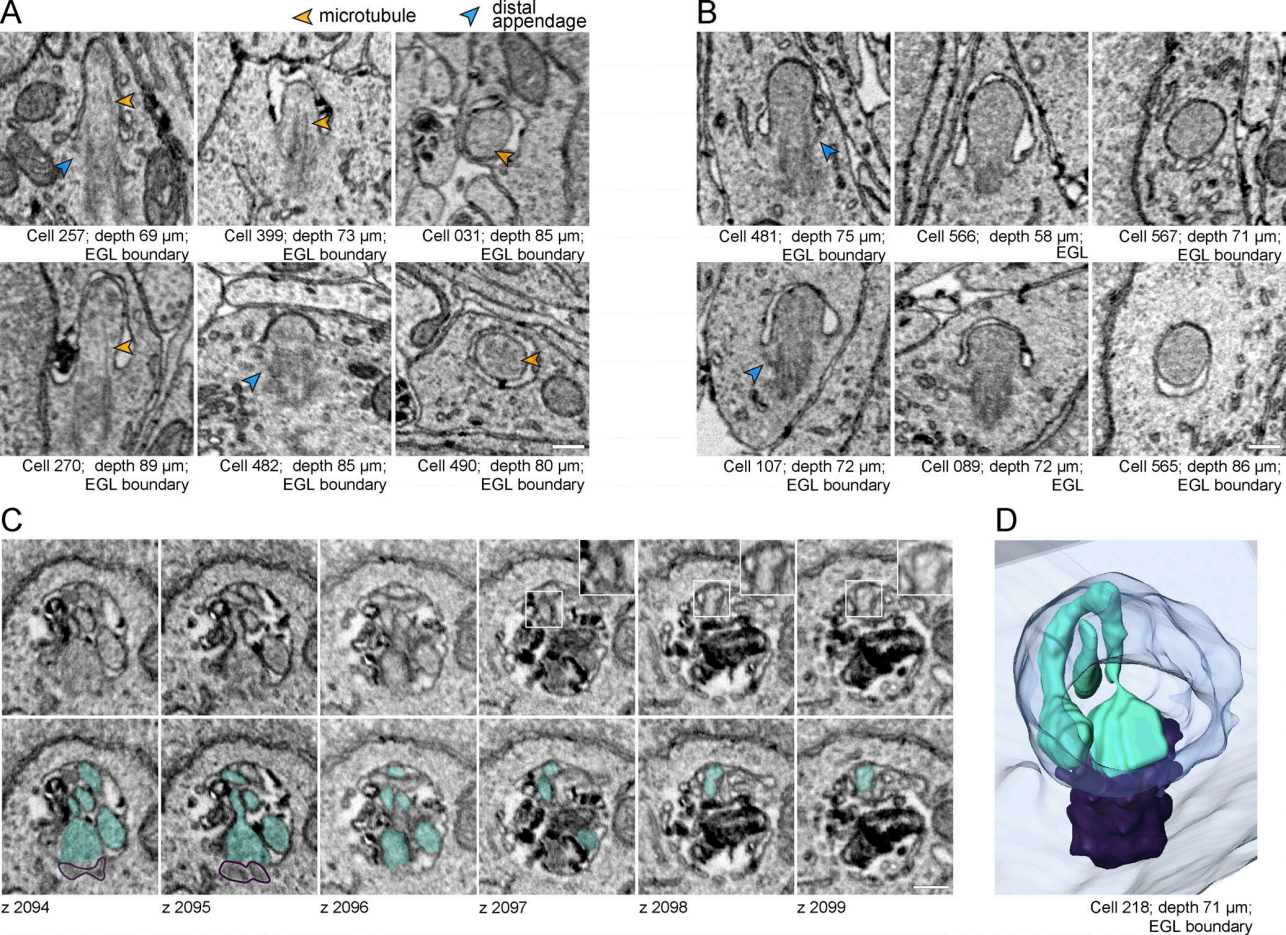
**Cilium deconstruction and docking intermediates. (A and B)** Representative images of short cilia with electron-lucent (A) or electron-rich (B) cilioplasm. Microtubules are highlighted with orange arrowheads and distal appendages with blue arrowheads. The scale bar is 200 nm. **(C and D)** A single cilium was observed with a constriction that could be indicative of cilium severing. Serial EM sections are shown in C. Microtubule singlets are visible in the insets on the top row and the cilium (and potential cilium fragment) are shaded cyan in the lower panel. A view of the 3D segmented image is presented in D.

To gain further insights into the process of cilia deconstruction in differentiating GC neurons, we closely examined cilium and centrosome ultrastructures within the EGL boundary. We recognized that a group of short, concealed cilia was distinct (Fig. 5 A and Fig. S3 A compared to Fig. 5 B and Fig. S3 B). Instead of tapering or rounding slightly at the tip like typical cilia, this subset had a rounded, bulbous shape. We labeled these as protein-rich cilia because the cilioplasm appeared more homogenous, amorphous, and darker than the adjacent cytoplasm or than the cilioplasm of other cilia, which indicated additional electron-dense staining. Axoneme microtubules and transition zones (Reiter et al., 2012) were difficult to discern in this subset of cilia either because they had depolymerized or because they were indistinguishable in the darker staining cilioplasm. We plotted the depth of the protein-rich cilia in comparison to the more typical short, concealed cilia (Fig. 5 C). While neither was restricted to a single layer, both were largely found in the EGL boundary, suggesting that protein-rich cilia were deconstruction intermediates observed during GC differentiation, but not during premitotic cilia resorption.

We searched for evidence of vesiculation from cilia or ciliary severing, established processes that shorten cilia (Cao et al., 2015; Esparza et al., 2013; Mirvis et al., 2019; Nager et al., 2017; Phua et al., 2017; Wang and Barr, 2016; Wang et al., 2015; Wood and Rosenbaum, 2014, 2015). We found only one possible example (Fig. S3, C and D). It was a concealed cilium with a constriction just above the base. If severing occurred the cilium would not be shed, but rather enclosed in a large intracellular vesicle. The infrequency of severing related structures indicates that more often short cilia arose from limited cilium growth in differentiating cells, and that disassembly in differentiating GCs involved a cilia deconstruction process, not cilia severing.

Upon examining cilia ultrastructure, we also noted that a subset of cilia contained internal vesicles (Fig. 5 D). These cilia were largely found in the EGL boundary; however, some cilia with internal vesicles were present in the more mature GCs of the IGL. Occasionally, protein-rich cilia contained internal vesicles. It seemed plausible that internal vesicles could have entered the cilium from the base; however, we found cilia with invagination of the ciliary membrane (Fig. 5 E), suggesting that internal vesicles were derived from invagination of the ciliary membrane. Endocytosis of the ciliary membrane could be a disassembly strategy that reduces the surface area of the cilium. While internal vesicles have been previously reported in chondrocytes (Jensen et al., 2004) and in specialized primary cilia, such as in olfactory neurons and in photoreceptors (Chuang et al., 2015; Jana et al., 2018; Reese, 1965), we are not aware of prior association with cilium disassembly.

**Figure 6.**
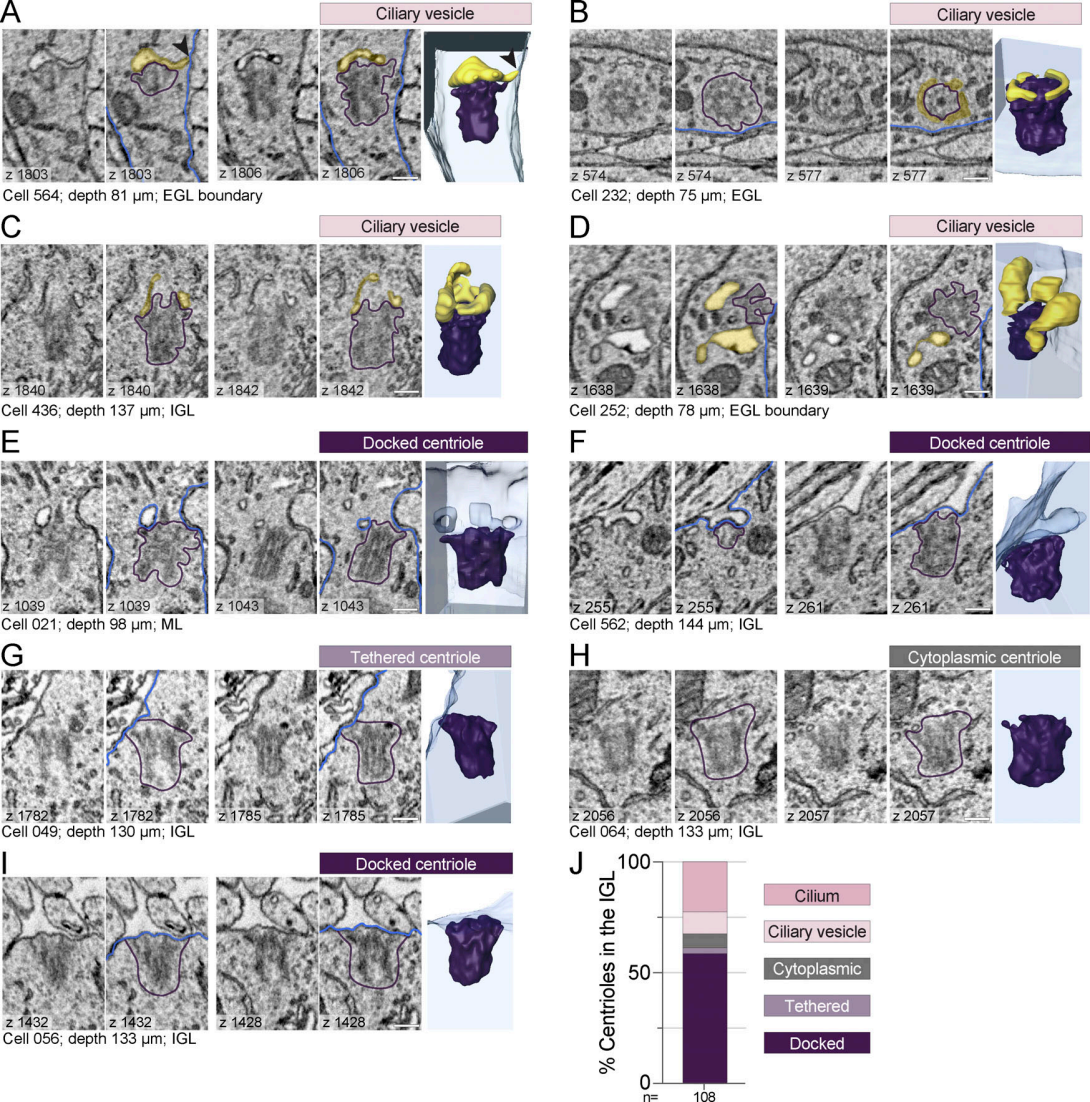
**Large-scale EM reveals novel cilia disassembly intermediates in differentiating GCs. (A–D)** Centrioles in differentiating GCs had ciliary vesicles. **(E and F)** Centrioles with plasma membrane invaginations attached to distal appendages. **(G and H)** Rare tethered centrioles (G), and cytoplasmic unciliated centrioles (H). **(I)** Unciliated mother centriole docked at the plasma membrane. For each centriole, raw and annotated representative EM sections are displayed to the left of the 3D reconstruction (yellow: ciliary vesicles; blue: cell boundary; purple: centriole). **(J)** The percentage of GCs in the IGL with centrioles in each classification are quantified. Scale bars are 200 nm.

The volumetric EM also provided unique views of centrioles in differentiating GC neurons. Throughout the cerebellar layers, but especially enriched in the EGL boundary, we found membrane structures associated with the mother centriole distal appendages (Fig. 6). Based on depth, we inferred which centrioles were likely in cycling progenitor cells and which were disassembly intermediates in differentiating cells (Fig. 5 A). Fig. 6 A (Video 5) shows a basal body in a GC at the EGL boundary capped with a ciliary vesicle and a short tube extending to the cell surface. During ciliogenesis, similar dynamic tubules project to the cell surface (Insinna et al., 2019). The structures in Fig. 6, B and C resembled preciliary toroid membranes (Videos 6 and 7) (Ganga et al., 2021; Insinna et al., 2019; Stuck et al., 2021); however, the membrane in Fig. 6 C extended up in places like a ciliary membrane. Fig. 6 D is an example of a centriole with multiple, large tethered vesicles. We also found unciliated centrioles that had invaginations of plasma membrane anchored to distal appendages (Fig. 6, E and F; and Video 8). We concluded that distal appendage-associated membranes in differentiating GCs in the EGL boundary, ML, PCL, and IGL are late-stage intermediates during cilia disassembly.

**Video 5.** **Centriole with ciliary vesicle in EGL boundary.** This z series includes the entire mother centriole of cell 564 from the EGL boundary of the P7 cerebellum shown in Fig. 6 A. 5 frames per second.

**Video 6.** **Centriole with toroid-shaped ciliary vesicle in EGL.** This z series includes the entire mother centriole of cell 232 from the EGL of the P7 cerebellum shown in Fig. 6 B. 2 frames per second.

**Video 7.** **Centriole with ciliary vesicle in IGL.** This z series includes the entire mother centriole of cell 436 from the IGL of the P7 cerebellum shown in Fig. 6 C. 3 frames per second.

**Video 8.** **Docked centriole with membrane invaginations in ML.** This z series includes the entire mother centriole of cell 021 shown from the ML of the P7 cerebellum in Fig. 6 E. 5 frames per second.

As we examined the centrioles in the IGL, we made another unanticipated discovery. We found unciliated mother centrioles anchored directly to the plasma membrane by their distal appendages. A few were tethered by the association of just a few distal appendages (Fig. 6 G and Video 9). Only a small subset of centrioles were immersed in the cytoplasm without any ciliary vesicles (Fig. 6 H). Most of these centrioles were fully docked, similar to the basal body of a surface cilium (Fig. 6 I and Video 10), and the distribution of ultrastructures found in the IGL is graphed in Fig. 6 J. The high frequency of docked centrioles suggests that docked centrioles are a hallmark end-state of cilia deconstruction during GC neurogenesis.

**Video 9.** **Tethered centriole in IGL.** This z series includes the entire mother centriole of cell 049 from the IGL of the P7 cerebellum shown in Fig. 6 G. 5 frames per second.

**Video 10.** **Docked centriole in IGL.** This z series includes the entire mother centriole of cell 056 from the IGL of the P7 cerebellum shown in Fig. 6 I. 5 frames per second.

### Unciliated mother centrioles dock at the plasma membrane in adult mouse GC neurons

**Figure 7.**
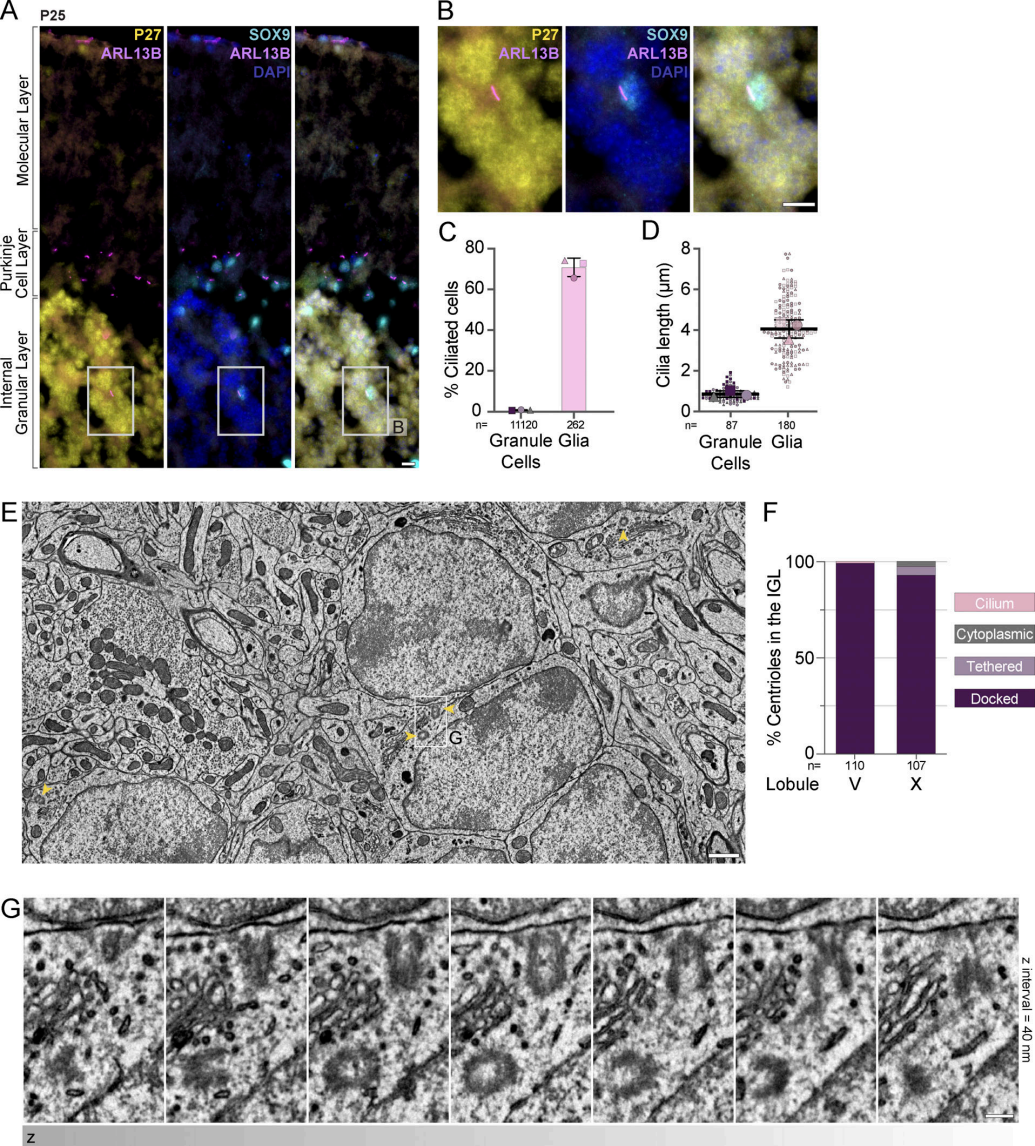
**Cilia are absent in mature GC neurons despite docking of mother centrioles at cell surface. (A)** Sagittal cerebella sections from a P25 mouse were immunostained with antibodies to ARL13B (magenta) to visualize cilia, P27^KIP1^ (yellow) to mark GC neurons, SOX9 (cyan) to mark glial cells, and counterstained with DAPI (blue) before imaging with widefield microscopy. Cilia are prominent in SOX9 glial cells; however, they are not detected on P27^KIP^ positive GC neurons. Scale bar: 10 μm. **(B)** Enlarged view of granule cells and a glial cell from the IGL of A. **(C and D)** Cilia frequency (C) and length (D) were measured in immunostained images acquired with widefield microscopy. Average frequencies of three sections each, from three animals is shown in C and individual cilia measurements from the same sections are shown as small symbols in D with the average per animal represented by the large symbol. The line and error bars represent the mean and standard deviation of the individual animal averages. **(E)** EM image from a serial-section transmission EM volume of the IGL of an adult mouse cerebellum. Centrioles are indicated with yellow arrowheads. Scale bar: 1 μm. **(F)** The percentage of GCs in the adult IGL with centrioles in each classification are quantified. **(G)** Serial sections of the centriole highlighted in E. Scale bar: 200 nm.

Centriole docking is an early step in the biogenesis of surface cilia (Breslow and Holland, 2019). The reported absence of cilia in adult GCs (Di Pietro et al., 2017), therefore, seemed at variance with the observed centriole docking. We sought to quantitatively evaluate cilia in the adult IGL. Toward this end, we examined sagittal sections of adult (P25) mouse cerebella with antibodies to ARL13B along with antibodies to P27^KIP1^ and SOX9 to positively identify GCs and glia, respectively (Farmer et al., 2016; Sun et al., 2017; Vong et al., 2015) (Fig. 7 A). We found that in the IGL <1% of GC neurons were ciliated, whereas 70% of glia (i.e., SOX9+ cells) were ciliated (Fig. 7, A and B). The cilia in the glial cells averaged ∼4 μm. By contrast, GC cilia averaged less than a micron in length by widefield fluorescence microscopy (Fig. 7, C and D). These results confirmed that mature GCs were largely unciliated.

**Figure S4.**
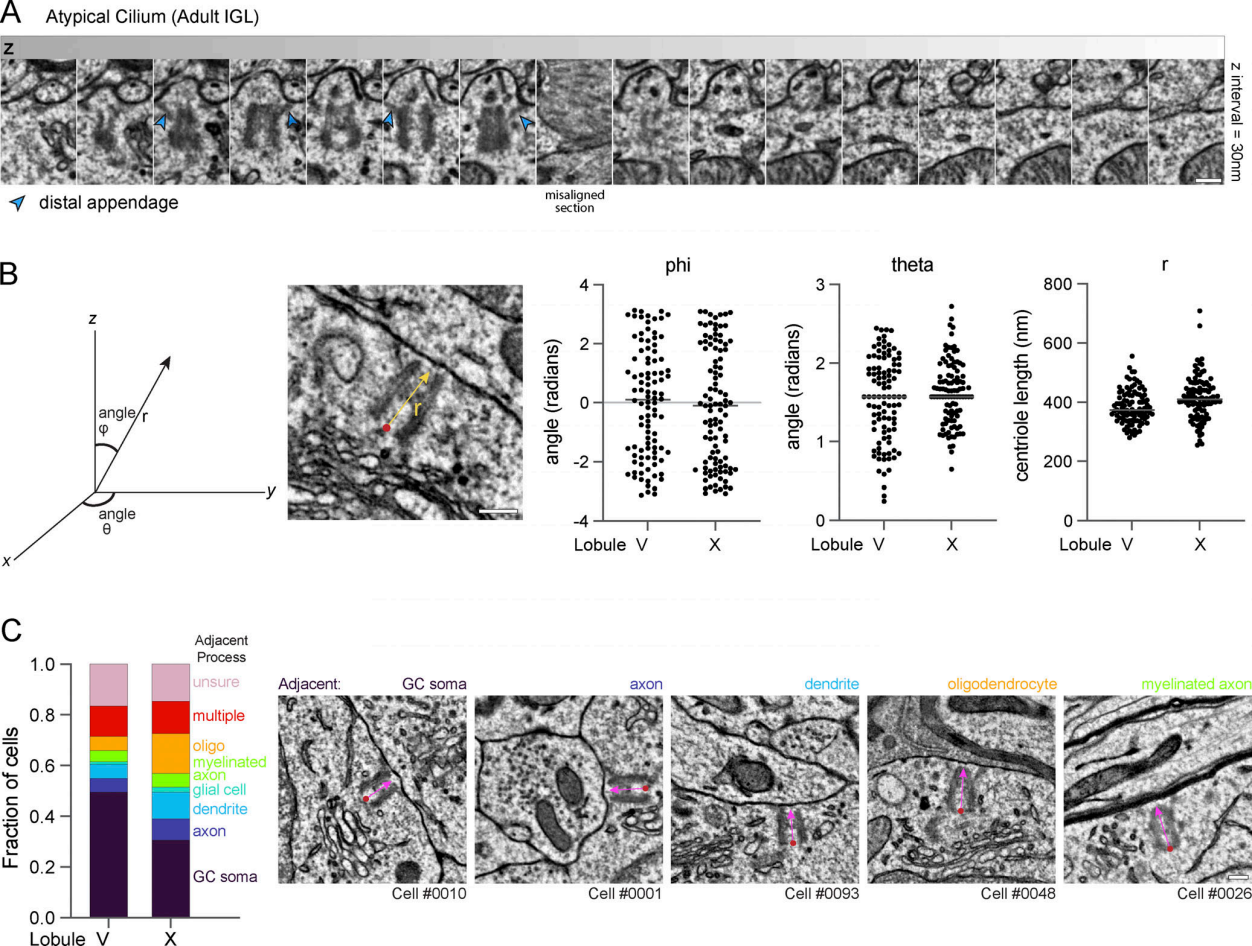
**Docked centrioles in adult GCs lack directed orientation. (A)** Serial EM sections display the only cilium annotated in the adult IGL volumes. Internal vesicles are present and the axoneme is not present or not resolved. Scale bar is 200 nm. **(B)** To assess the polarity of centriole docking, we generated a vector from proximal to distal within each mother centriole. We compared the vectors and found no bias in the orientation of docked centrioles. **(C)** The type of structure immediately adjacent to the plasma membrane where each GC mother centriole docked was determined. The distributions for the annotated centrioles in each adult dataset are plotted on the left and EM images on the right provide examples of centrioles docked adjacent to the indicated structures.

To determine whether the unciliated centrioles in adult GC neurons are docked, we utilized two recently generated EM volumes created to investigate synaptic connections (Nguyen et al., 2023). We located, annotated, and analyzed >100 centrosomes in each of two datasets of serial-section transmission EM from lobule V and lobule X of the adult mouse vermis imaged at a resolution of 4.3 × 4.3 × 45 nm (Nguyen et al., 2023; unpublished data). Almost every mother centriole was attached to the plasma membrane through the adhesion of the distal appendages (Fig. 7, E–G). We only found a single, short cilium with internal vesicles in the lobule V volume (Fig. 7 F and Fig. S4 A) and five tethered and three cytoplasmic centrioles in the lobule X volume (Fig. 7 F). In combination with the analysis of docked centrioles in the P7 IGL, we conclude that GC neurons in adult mice have largely lost their cilia by a pathway involving cilia deconstruction and centriole docking during GC neural differentiation.

We investigated the cellular context of the docked centrioles in search of functional insights. Centrioles docked at the plasma membrane in the small region of each GC in areas where Golgi, mitochondria, and most other organelles were concentrated. Ciliary rootlet structures were visible in many GCs. We measured the orientation of centrioles and found no positioning or orientation bias within the tissue (Fig. S4 C). We also determined the type of structures extracellular to each mother centriole. Unlike T cells which transiently dock mother centrioles at the immune synapse to direct release of lytic granules toward a target cell (Stinchcombe et al., 2015), we found diverse structures opposite the GC docked centrioles including the soma of other GCs, axons, dendrites, and glial processes including myelin sheaths (Fig. S4 C), and no evidence of centriole nucleated microtubules forming highways positioned to deploy vesicles. Instead of being oriented relative to external factors, centriole docking appeared to influence the internal organization of the GC soma. Although centriole docking can be the first stage in the biogenesis of surface cilia, during differentiation, GCs disassemble cilia, anchor the centriole to the plasma membrane, and remain unciliated.

## Discussion

Primary cilia are required for the proliferation of GC neural progenitors (Chizhikov et al., 2007; Spassky et al., 2008). We discovered that shortened cilia present early in differentiation disassembled as GC neurons matured and did not regrow despite mother centrioles docking like basal bodies at the cell surface. The lack of cilia in GC neurons is remarkable because most other neuronal subtypes have cilia (Green and Mykytyn, 2014; Louvi and Grove, 2011; Ott et al., 2024; Wu et al., 2024). We detected previously undescribed disassembly intermediates with interesting similarities to structures observed during ciliogenesis (Ganga et al., 2021; Insinna et al., 2019; Stuck et al., 2021). To our knowledge, this is the first ultrastructural description of cilia disassembly in postmitotic, differentiating cells. To distinguish it from premitotic cilia resorption, we refer to cilia disassembly in differentiating GCs as cilia deconstruction.

The concealed cilia we observe could be intermediates in the internal ciliogenesis pathway (Sorokin, 1962, 1968) or could be reversibly generated from surface-exposed cilia, as reported in cultured cells (Rivera-Molina et al., 2021). The prevalence of concealed primary cilia could not have been predicted from light microscopy images. Also reminiscent of intracellular ciliogenesis intermediates, we found that some centrioles in maturing GC neurons had distal appendage–associated membranes (Breslow and Holland, 2019; Zhao et al., 2022). However, we were able to infer that the observed cilia intermediates were disassembling, not assembling, due to their location and the correlation between cell position and developmental status in the cerebellum. The presence of concealed cilia and the observed similarities suggest that the mechanisms used to build and maintain cilia might influence the deconstruction of preformed cilia (Constable et al., 2023, *Preprint*).

**Figure 8.**
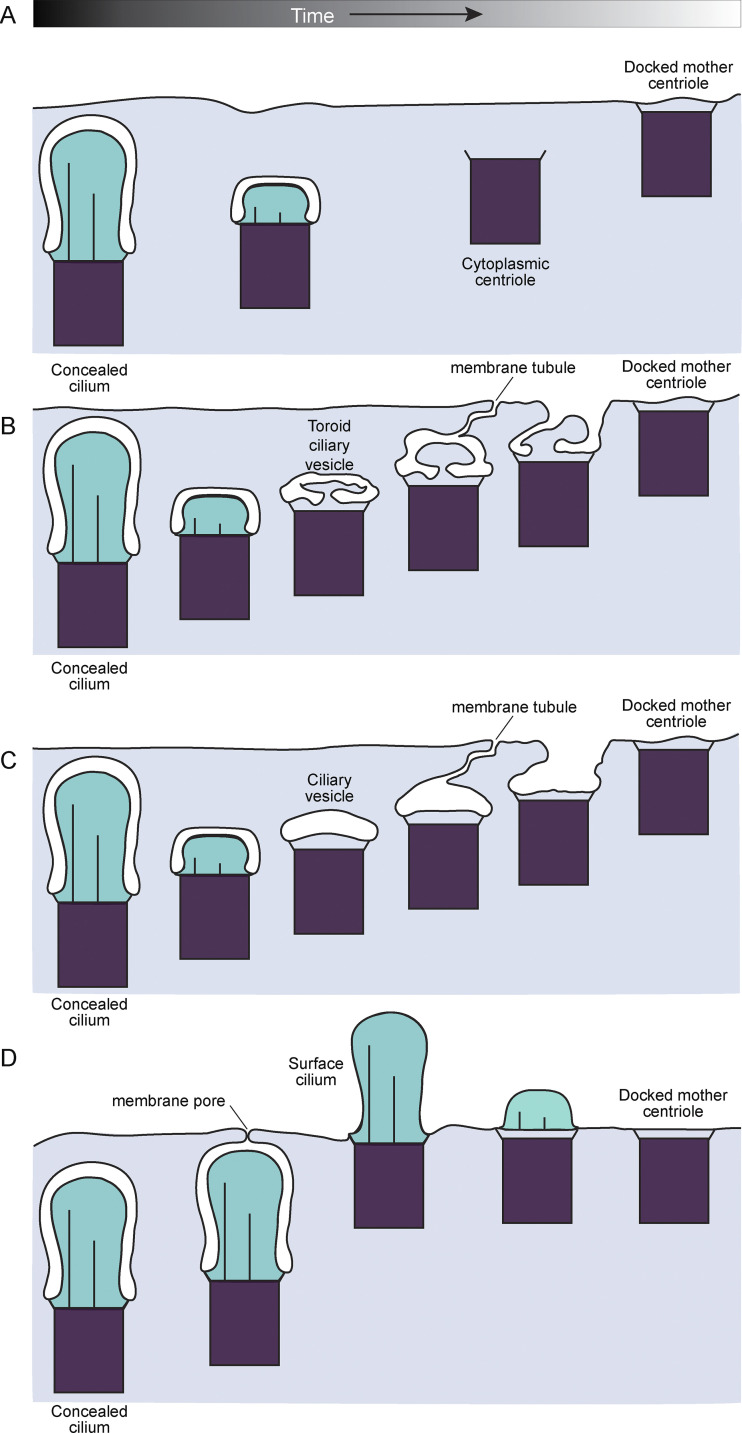
**Centriole docking initiated at different stages in cilia deconstruction could account for the variety of intermediates observed.** Differences between late-stage cilia/centrosome structures in differentiating cells suggest that instead of a linear deconstruction pathway, variance in the coordination of cilia deconstruction and mother centriole docking could generate multiple routes to mature, unciliated cells with docked mother centrioles. **(A)** Complete cilia deconstruction results in cytoplasmic centrioles, which then dock at the plasma membrane similar to centriole docking during surface cilia biogenesis and at the immune synapse. **(B and C)** Cilia deconstruction includes centriole associated membrane intermediates including ciliary vesicles (B) or toroid membranes (C). Centriole docking commences utilizing dynamic tubules to unite the ciliary vesicle with the plasma membrane. **(D)** Concealed cilia could access the plasma membrane through observed membrane pores. Opening of the pore before the cilium has been deconstructed could result in surface cilia, which subsequently get completely disassembled.

The shapes of centriole-docked membranes were especially diverse, possibly due to membrane dynamics not captured in the single EM volume. Unlike in premitotic cells, we were unable to assemble a single linear disassembly pathway from the observed disassembling structures in differentiating GCs. We propose that the heterogeneity of the observed intermediates reflected diversity in the cilia deconstruction and centriole docking pathway. In Fig. 8, we have illustrated four possible progressions of cilia deconstruction which include different observed intermediate structures that could all resolve as docked centrioles. Several scenarios hypothesize that membrane pores or tubules, similar to the dynamic PACSIN- and EHD1-dependent tubules involved in ciliogenesis (Ganga et al., 2021; Insinna et al., 2019; Stuck et al., 2021), join the internal cilia membrane to the plasma membrane. The difference between each model is the extent of cilia disassembly and the presence of anchored vesicles prior to centriole docking. First, we show the conventional paradigm of a cytoplasmic mother centriole docking directly to the plasma membrane. The second and third illustrations involve the fusion of distal appendage anchored vesicles to the plasma membrane resulting in plasma membrane invaginations that would be resolved to generate docked centrioles. Finally, fusion of the ciliary sheath before complete cilia deconstruction results in surface cilia similar to those observed in the IGL (many of which had atypical morphology, lacked axonemes, or contained intraciliary vesicles). Live monitoring of cilia deconstruction and centriole docking will be necessary to determine if one or all of these docking pathways occur within the tissue.

The newly described cilia deconstruction pathway appears to include different intermediates than those seen in ciliary disassembly that occurs prior to cell division (Pugacheva et al., 2007; Tucker et al., 1979) or during the post-fertilization stage of *Chlamydomonas* (Pan and Snell, 2003, 2005; Pan et al., 2004). Thus, our results suggest that established cilia disassembly mechanisms (Liang et al., 2016; Malicki and Johnson, 2017; Wang and Dynlacht, 2018) may not fully account for cilia loss during GC differentiation. We find no evidence for the rapid disassembly of microtubules (Pan and Snell, 2005; Pugacheva et al., 2007), internalization of a portion of the axoneme (Rieder et al., 1979), or shear stress (Liang et al., 2016), which have all been shown to contribute to cilia disassembly in other systems. The persistence of a few cilia in postnatal IGL GCs beyond the 48 h BrdU pulse indicates a wide spatiotemporal distribution of deconstructing cilia during GC differentiation. The process of cilia deconstruction involves gradual axoneme depolymerization and recovery of the ciliary membrane, as suggested by enhanced electron-dense staining in protein-rich cilia and internal cilia vesicles. In addition, the observed late-stage centriole-associated vesicles indicate that ciliary membrane and ciliary pocket retrieval are coordinated in the final stages.

Developmental and circadian-regulated cilia disassembly followed by regrowth has been reported in diverse systems (Das and Storey, 2014; Lepanto et al., 2016; Toro-Tapia and Das, 2020; Tu et al., 2023). During GC neurogenesis, however, not only did the mother centrioles in adult GCs remain unciliated but they docked at the plasma membrane. Because centriole docking is pervasive in adult GC neurons, it is likely that centriole docking is persistent. Such docking was unexpected because there are only a few systems where centriole docking without cilia extension has been described, which includes the immune synapse (Stinchcombe et al., 2015). Mother centrioles in T cells transiently dock at the plasma membrane adjacent to the target cell. Unlike docked centrioles at the T cell synapse, docked cerebellar GC centrioles do not appear to create a hotspot for exocytosis. We found additional evidence for centriole docking in historic electron micrographs of GC neurons (Del Cerro et al., 1969) and gamma cells in the adrenal cortex (Wheatley, 1967).

The changes in cilia and centrosome transcripts and proteins that accompany GC differentiation have recently been investigated. Global, developmentally programmed, diminution of cilium maintenance, rather than active disassembly, causes cilia deconstruction in differentiating GCs (Constable et al., 2023, *Preprint*). In addition, centriole capping proteins, which prevent ciliogenesis, assemble onto mother centrioles as GC neurons mature (Constable et al., 2023, *Preprint*), similar to the immune synapses in T cells (Stinchcombe et al., 2015). These results might explain why docked centrioles, which could develop surface cilia, remain unciliated.

The process of cilia concealment and deconstruction described here may be important to prevent aberrant SHH signaling. Soluble SHH secreted by Purkinje neurons permeates the EGL, yet upon onset of differentiation, GCs stop responding to the mitogenic signal (Corrales et al., 2004). We hypothesize that retention of concealed cilia early in differentiation facilitates SHH pathway suppression through the formation of GLI transcriptional repressor (Han et al., 2009; Kopinke et al., 2020). Loss of SHH pathway inhibition in neural stem cells and GCs from lack of GPR161 and SUFU, which promotes cleavage of GLI3 into the transcriptional repressor GLI3R, can lead to hyperproliferation and cerebellar dysplasia (Blaess et al., 2008; Jiwani et al., 2020; Shimada et al., 2018). Thus, we speculate that cilia-localized GPR161, as well as downstream adenylyl cyclases, could function through concealed cilia to suppress SHH pathway–mediated hyperproliferation (Shimada et al., 2018; Somatilaka et al., 2020). Concealment could also simultaneously ensure that proliferative programming is not reactivated during neuronal maturation by SHH detection (Han et al., 2009; Kopinke et al., 2020). Subsequent cilia deconstruction may be important in adult tissue. One prevalent subtype of medulloblastoma, a cerebellar brain tumor, is caused by the unrestricted proliferation of GCs with progenitor characteristics (Schuller et al., 2008; Yang et al., 2008) from aberrant activation of the SHH signaling pathway (Oliver et al., 2005; Shimada et al., 2018). The GCs in SHH-medulloblastoma can be ciliated (Di Pietro et al., 2017; Han et al., 2009; Youn et al., 2022). In mice embryos, a docked centriole is sufficient to provide cues to maintain renal tubule architecture, but postnatally a cilium is required for tubular homeostasis (San Agustin et al., 2016). However, lack of cilia formation from the docked centrioles in adult GCs likely suppresses SHH receptivity, blocking proliferative potential and dedifferentiation. Our results suggest that both proper deconstruction of cilia and prevention of cilia regrowth are needed to permanently disable SHH signaling in GC neurons.

Cilia loss during differentiation is not unique to GC neurogenesis. Adipocytes (Hilgendorf et al., 2019; Zhu et al., 2009), retina pigment epithelial cells (Patnaik et al., 2019), myoblasts (Fu et al., 2014), steroidogenic adrenal cortical cells (King et al., 2009; Mateska et al., 2020), and oligodendrocytes (Buchanan et al., 2022) are all derived from ciliated progenitor cells. For example, in the visual cortex, the oligodendrocyte precursor cells (OPCs) that give rise to the non-ciliated oligodendrocytes have cilia (Ott et al., 2024). Interestingly, some of the cilia in OPCs are similar to the concealed cilia and show ciliary vesicles similar to the differentiating GC neurons (Ott et al., 2024). Hypertrophic chondrocytes also have lower ciliation compared with the columnar chondrocyte precursors (Hwang et al., 2018). Cilia absence could be caused by prevention of cilia re-growth after pre-mitotic cilia resorption in the precursors; however, in some of these diverse contexts, the cilia deconstruction pathway we identified may be responsible for cilia loss and altered physiology. Although cilia are pervasive in many tissues, our study highlights the equal importance of eliminating cilia in defined developmental contexts.

## Materials and methods

### Mouse handling and genotyping

All animal studies were approved in accordance with UTSW Institutional Animal Care and Use Committee regulations and were conducted in accordance with NIH guidelines for the care and use of laboratory animals. CD1 mice were purchased from Charles River Laboratories and maintained under standard conditions. Mice were treated with 10 mg/ml BrdU (Bromodeoxyuridine/5-bromo-2′-deoxyuridine) dissolved in PBS using intraperitoneal injection at 50 mg/kg.

### Mouse brain processing

Mice were procured at the appropriate age and fixed by transcardial perfusion using 4% paraformaldehyde (PFA) in PBS after appropriate anesthesia for their age (either isoflurane or cold exposure on ice) according to IACUC regulations. Brains were removed and further fixed in 4% PFA/PBS overnight at 4°C on a rotator and then immersed in 30% sucrose in PBS until the brain sank to the bottom of the tube (∼48 h). Brains were cut in half in the sagittal direction and embedded cut face down in cryomolds using OCT embedding media (BioTek) and frozen on dry ice until solid. Blocks were stored at −80°C until sectioning on a Leica Cryostat model CM1950 cryostat at 15–30 μm thickness. Sections were stored at −20 or −80°C until staining.

### Immunofluorescence staining and light microscopy

Sections were thawed at room temperature and OCT was removed by immersion in PBS. Sections were blocked using 3% serum (donkey) in PBS with 0.3% Triton-X 100 for 30 mins. Primary antibodies were diluted in blocking solution at the appropriate dilutions and incubated overnight at room temperature in a humid chamber. Primary antibodies: ARL13B (1:1,000, #75-287; UC Davis/NeuroMab), BrdU (1:500, #ab6326; Abcam), P27^KIP^ (1:400, #610241; BD Biosciences,), and SOX9 (1:500, #ABE571; Millipore). For BrdU staining, we performed 2 N HCl pretreatment for 15 min at 37°C before the blocking solution was applied. Sections were incubated with the indicated isotype-specific secondary antibodies for 2 h at room temperature. Sections were washed three times, and DAPI (1 μg/ml) Sigma-Aldrich or Hoechst 33342 (Invitrogen) was included in the final wash to stain nuclei. Stained tissues were mounted using Fluromount-G (Southern Biotech) and allowed to dry overnight. Stained slides were imaged within 2–3 days and stored at 4°C (short term) or −20°C (long term).

Images were acquired on a widefield microscope (AxioImager.Z1; ZEISS), confocal microscope (LSM880; Zeiss), or a spinning disk confocal microscope (Nikon CSU-W1 SoRa). Images in the widefield microscope were acquired using a Plan Apochromat objective (40×/1.3 NA oil and 63×/1.4 NA oil) and sCMOS camera (PCO Edge; BioVision Technologies) controlled using Micro-Manager software (University of California, San Francisco) at room temperature. Images in the confocal microscope (LSM880; Zeiss) were acquired using Plan Apochromat objective (63×/1.4 NA oil). Images in the spinning disk confocal microscope (Nikon CSU-W1 SoRa) were acquired using a Plan Apochromat objective (100×/1.45 NA oil), a sCMOS camera (Hamamatsu Orca-Fusion), and a Piezo z-drive for fast z-stack acquisition controlled using Nikon NIS-Elements software at room temperature. Between 10 and 30 z sections at 0.2-µm intervals were acquired.

### Image analysis

#### Cilia length and number determination

Images were analyzed using Fiji (Schindelin et al., 2012). Different cerebellum layers were identified using nuclei, and P27^KIP^ markers. Cilia length was manually determined by tracing in each zone at zoom level 200–300% using the freehand draw tool, and measurement was recorded using a measure tool. Cilia already traced were permanently marked with a draw tool to ensure unique cilia were measured when moving around the image. Zones were completed in their entirety before moving on to the next zone. The number of cilia was determined by counting the number of cilia measured. The number of cells was determined by counting the total number of nuclei found in each section as stained by Hoechst or DAPI.

### Annotation of EM data

Each EM volume (Nguyen et al., 2023; Wilson et al., 2019) was uploaded onto a CATMAID (Saalfeld et al., 2009) server. Centrioles and cilia were manually located and annotated. Centrioles were marked at the center inside the distal end of the centriole and a sphere with a 500-nm radius was placed at the node for 3D visualization. The initial node of the cilia skeleton was placed at the center of the base of the cilium (which is also the proximal end of the centriole). Subsequent cilia nodes tracked the center of the cilium, and a tag was added to the skeleton to mark the location where a pocket cilium exited the cell. Centriole vectors in the adult volumes originated at the center of the distal end of the cilium and terminated at the center of the proximal end (Fig. S4 B). The classifications used for annotating in the P7 and the adult volumes are listed in Table S2. Although it is a rough approximation, in the P7 volume, layer boundaries do not correlate directly with cell depth because of the orientation of the sample during EM sectioning. Layer annotations for each cell were therefore manually determined by the immediate cell context. The code used to extract data from the CATMAID annotations can be found at: https://github.com/pattonw/centriole_data.

### Visualization and segmentation of EM data

Images and volumes were cropped from the EM volume in CATMAID. Images were rotated to similarly orient all centrioles. Where needed to improve image quality or to compensate for differences within the raw data, image brightness and contrast were adjusted using Fiji/ImageJ. For segmentation, image stacks were imported into Amira. Each structure was segmented in a separate layer before meshworks were generated and the images of the 3D structure were captured. Segmentation was approximated where image quality was uninterpretable.

### Graphing and statistics

All graphs were generated using Prism. Superplots were generated by overlaying average values from each animal into individual values, as explained in Lord et al. (2020). Statistical significance was determined in Prism using ordinary one-way ANOVA using multiple comparison analysis with Turkey correction. Population mean was assessed at a 95% confidence interval and was considered significant at the following P values: 0.0332 (*), 0.0021 (**), 0.000-2 (***), and <0.0001 (****).

### Online supplemental material

Fig. S1 shows the distribution of cilia lengths for the entire annotated dataset and broken down by cilium type (pocket, concealed, surface). It also includes the depth and cilium type distribution for cells in G1/G0 and cells in S/G2. Fig. S2 shows a z-stack progression and a 3D reconstructed example of a cilium that is enveloped by another cell immediately upon exiting the ciliary pocket. Fig. S3 shows examples of short concealed cilia with electron-rich or electron-lucent cilioplasms. In addition, the z stack and 3D reconstruction of a cilium potentially being severed is provided. Fig. S4 shows a cilium in the adult IGL and quantitation of docked mother centriole orientation and adjacent structures in the adult IGL. Videos 1, 2, 3, and 4 are z series that include the entire cilia of cells 451, 444, 518, and 368 shown in Fig. 4. Videos 5, 6, 7, 8, 9, and 10 are z series of each cilium/centriole shown in Fig. 6 from cells 564, 232, 436, 021, 049, and 056. Table S1 provides details about the location and features of each annotated centriole/cilium. Table S2 has two tabs. The first describes the annotation classifications used for the P7 dataset and the second tab describes the annotation classification used in the adult datasets.

## Supplementary Material

Table S1 provides details about the location and feature of each annotated centriole/cilium.

Table S2 has two tabs. The first describes the annotation classifications used for the P7 dataset and the second tab describes the annotation classification used in the adult datasets.

## Data Availability

Published datasets used in this study are available from original publications. All other data are available from the corresponding author upon request.
